# Bushen Huoxue formula attenuates lipid accumulation evoking excessive autophagy in premature ovarian insufficiency rats and palmitic acid-challenged KGN cells by modulating lipid metabolism

**DOI:** 10.3389/fphar.2024.1425844

**Published:** 2024-09-16

**Authors:** Tian Li, Yao Wei, Beibie Jiao, Rui Hao, Beibei Zhou, Xinlan Bian, Peijuan Wang, Yahong Zhou, Xia Sun, Jian Zhang

**Affiliations:** ^1^ Nanjing Lishui District Hospital of Traditional Chinese Medicine, Nanjing, China; ^2^ Clinical College of Traditional Chinese Medicine Hospital in Lishui, Jiangsu Health Vocational College, Nanjing, China; ^3^ Affiliated Hospital of Integrated Traditional Chinese and Western Medicine, Nanjing University of Chinese Medicine, Nanjing, China; ^4^ Tonglu Hospital of Traditional Chinese Medicine, Tonglu, China; ^5^ Wuxi Hospital Affiliated to Nanjing University of Chinese Medicine, Wuxi, China

**Keywords:** premature ovarian insufficiency, bushen huoxue formula, lipid, ROS, autophagy

## Abstract

**Introduction:**

Premature ovarian insufficiency (POI) has affected about 3.7% of women of reproductive age and is a major factor contributing to infertility. Bushen Huoxue formula (BHF), a traditional Chinese medicine prescription, is clinically used to treat POI in China. This study aims to investigate the potential mechanisms of BHF in combating POI using corticosterone-induced rats and palmitic acid (PA)-challenged human ovarian granulosa cells (GCs).

**Methods:**

Initially, ultra performance liquid chromatography tandem mass spectrometry was employed to analyze the components of BHF. The pharmacodynamic parameters evaluated included body weight, ovaries index, and serum hormone in rats. Follicle numbers were observed using H&E staining. Additionally, PCNA and TUNEL staining were used to assess GCs proliferation and apoptosis, respectively. Lipid accumulation and ROS levels were examined using Oil Red O and ROS staining. Protein expressions were determined by western blot. To probe mechanisms, cell viability and E_2_ levels in BHF-treated, PA-stimulated GCs were determined using MTT and ELISA, respectively. Cell apoptosis and ROS levels were assessed using TUNEL and ROS staining. Proteins related to lipid metabolism and autophagy in PA-stimulated GCs were studied using agonists.

**Results:**

Our results shown that BHF effectively normalized serum hormone levels, including follicle-stimulating hormone (FSH), anti-Müllerian hormone (AMH), estradiol (E_2_), and luteinizing hormone (LH). Concurrently, BHF also significantly reduced follicular atresia and promoted cell proliferation while inhibiting apoptosis in POI rats. Furthermore, BHF mitigated ovarian lipid accumulation by modulating lipid metabolism, which included reducing lipid synthesis (expression of peroxisome proliferator-activated receptor γ and CCAAT/enhancer binding protein α), increasing lipid catabolism (expression of adipose triglyceride lipase), and enhancing lipid oxidation (expression of carnitine palmitoyl transferase 1A). Mechanistically, the therapeutic effects of BHF on POI were linked with alleviation of lipid deposition-induced reactive oxygen species (ROS) accumulation and excessive autophagy, corroborating the results in PA-challenged GCs. After treatment with elesclomol (a ROS inducer) and rapamycin (an autophagy inducer) in GCs, the effects of BHF were almost counteracted under model conditions.

**Conclusion:**

These findings suggest that BHF alleviates the symptoms of POI by altering lipid metabolism and reducing lipid accumulation-induced ROS and autophagy, offering evidence for BHF’s efficacy in treating POI clinically.

## 1 Introduction

Premature ovarian insufficiency (POI) is characterized by a decline in ovarian function before the age of 40, affecting approximately 3.7% of women of reproductive age globally (Golezar et al., 2019). Diagnosis is based on clinical features including menstrual irregularities or amenorrhea, elevated gonadotrophins (FSH level > 25 IU/L on two separate tests at least 4 weeks apart), and reduced estradiol (E_2_) levels (Ishizuka, 2021). Studies have shown that the natural pregnancy rate in women with POI is below 5%, making it a significant factor in infertility among women of reproductive age (Bidet et al., 2011). Furthermore, POI is associated with increased risks of neurological dysfunction, type 2 diabetes, cardiovascular diseases, osteoporosis, and reduced life expectancy (Tucker et al., 2016).

Current evidence suggests that POI etiologies are linked to environmental factors, iatrogenic injuries, genetic defects, autoimmune dysfunctions, and metabolic abnormalities (Huhtaniemi et al., 2018; Jiao et al., 2021; Wang, et al., 2020b). These complex exogenous and endogenous factors contribute to abnormal follicle activation, hindered recruitment of dominant follicles, impeded maturation, and increased follicular atresia, ultimately leading to POI (Li Z. et al., 2021). Apoptosis in granulosa cells (GCs) can initiate follicle atresia and lead to oocyte loss, which is a critical factor in POI development (Li D. et al., 2021). Prior research on GC apoptosis has focused on reactive oxygen species (ROS) generation, cytokine, and hormone disruptions (Matsuda et al., 2012). However, recent studies show that imbalances in lipogenesis and lipolysis, causing lipid deposition and subsequent lipotoxicity and ROS generation, are key in GCs apoptosis (Shaoyong et al., 2022). In POI patients, triglyceride (TG) and free fatty acid (FFA) levels are significantly elevated in follicular fluid compared to healthy individuals (Huang et al., 2022; Wang L. et al., 2020). High FFA levels in follicular fluid have been shown to suppress GCs proliferation, as observed in dairy cows (Wang Y. et al., 2020). Additionally, FFAs induce apoptosis in GCs (human primary GCs and human KGN cells) in a dose-dependent manner (Baddela et al., 2020) and elevate ROS content (Ma et al., 2022), which inhibits proliferation and promotes senescence in GCs by triggering autophagy (Sun et al., 2021). Thus, reducing lipid deposition emerges as a potential therapeutic approach for POI.

While hormone replacement therapy (HRT) is commonly used for treating POI in clinical settings, it often results in limited clinical effectiveness or adverse side effects (Webber et al., 2017). HRT is not reported to aid in follicular development or reverse ovarian function decline (Li et al., 2020). Recent studies have shown that Traditional Chinese Medicines may delay ovarian failure and have a high safety profile in POI treatment (Li et al., 2020). Bushen Huoxue formula (BHF), a traditional clinical prescription, is widely used in China for ovarian failure-related diseases (Zhongwei et al., 2019). Our previous study indicated that BHF significantly enhanced ovarian function and restored triglyceride homeostasis in psychological stress-induced POI rats (Miao et al., 2020). Interestingly, BHF also showed efficacy in regulating lipid metabolism and reducing ROS in diabetic retinopathy rats and nucleus pulposus cells (Gao et al., 2022; Wang et al., 2020c; Li, et al., 2020). However, the specific role of BHF in treating POI by improving lipid levels remains unclear.

In this context, we hypothesize that BHF treatment in POI may rectify impaired lipid deposition, thereby reducing ROS and autophagy. To explore this, we employed FFA-stimulated GCs and corticosterone-induced rats to investigate BHF therapeutic effectiveness and the underlying mechanisms in POI treatment.

## 2 Materials and methods

### 2.1 Drugs and reagents

BHF consists of eleven medicinal herbs (Table 1) which were purchased from Nanjing Lishui District Hospital of Traditional Chinese Medicine (Nanjing, China). Progynova was provided by DELPHARM Lille S.A.S. Corticosterone was purchased from Shanghai Aladdin Bio-Chem Technology Co., Ltd. (Shanghai, China). Estradiol (E_2_) and follicle-stimulating hormone (FSH) ELISA kits were obtained from the Elabscience Biotechnology Co., Ltd. (Wuhan, China). Corticosterone, Luteinizing Hormone (LH), and anti-Müllerian hormone (AMH) ELISA kits were from the Wuhan Xinqidi Biotech Co., Ltd. (Wuhan, China). BCA protein assay kits were bought from Beyotime (Shanghai, China). Primary antibodies against PPARγ, c/EBPα, ATGL, and CPT1A were supplied by Proteintech (Chicago, United States). LC3B and p62 were purchased by ABclonal (Wuhan, China).

**TABLE 1:** The components of BHF.

Chinese name	Latin name	Lot number	Dosage (g)	Occupied percent (%)
Sheng Di Huang	*Rehmannia glutinosa* Libosch.	220401	10	9.43
Yin Yang Huo	*Epimedium brevicornu* Maxim.	211001	10	9.43
Tu Si Zi	*Cuscuta chinensis* Lam.	A220616	10	9.43
Gou Teng	*Uncaria rhynchophylla (Miq.) Miq. ex* Havil.	A220223	10	9.43
Dang Gui	*Angelica sinensis* (Oliv.) Diels.	A220706	10	9.43
Bai Shao	*Paeonia lactiflora* Pall.	A220513	10	9.43
Chuan Xiong	*Ligusticum chuanxiong* Hort.	220601	10	9.43
Zhi Mu	*Anemarrhena asphodeloides* Bge.	220402	10	9.43
Chai Hu	*Bupleurum chinensis* DC.	211130	6	5.66
Huang Bai	*Phellodendron chinensis* Schneid.	210928	10	9.43
Mu Dan Pi	*Paeonia suffruticosa* Andr.	220402	10	9.43

### 2.2 Preparation and qualitative analysis of BHF

BHF was prepared using a previously established method. Initially, In our experiment, all herbs were soaked in distilled water at ten times their volume (v/m) for 30 min at 24°C, followed by decoction for an additional 30 min at 100°C. The mixture was then filtered through gauze. The herb residue was re-extracted under identical conditions. The two filtrates were consolidated and concentrated under depressurized conditions, followed by lyophilization to powder for qualitative analysis and subsequent experiments.

For the analysis, 500 mg of BHF powder was immersed in 1 mL of 70% methanol and ground for 3 min using an automatic sample rapid grinder (jxfstprp-48, 70 Hz). The solution was then centrifuged at 12,000 rpm for 10 min at 4°C and filtered through a 0.22 μm PTFE filter. UPLC-MS/MS was used for analysis. UPLC was performed on a Thermo Vanquish UHPLC using a Zorbax Eclipse C18 (1.8 μm × 2.1 mm × 100 mm) column at 30°C, with pure acetonitrile as solvent A and 0.1% formic acid as solvent B. The gradient elution program was as follows: 5% A at 0–2 min, 5%–30% A at 2–6 min, 30% A at 6–7 min, 30%–78% A at 7–12 min, 78% A at 12–14 min, 78%–95% A at 14–17 min, 95% A at 17–20 min, 95%–5% A at 20–21 min, and 5% A at 21–25 min. The flow rate was set at 0.3 mL/min, with an injection volume of 2 μL. Mass spectrometry was conducted on a Q-Exactive HF from Thermo Fisher in both positive and negative ionization modes. The parameters were: heater temperature at 325°C; sheath gas flow at 45 arb (arbitrary units); electrospray voltage at 3.5 KV; capillary temperature at 330°C; S-Lens RF level at 55%; full scan (m/z 100–1,500) and data-dependent MS2 (TopN = 10); resolutions at 120,000 (MS1) and 60,000 (MS2).

### 2.3 Animals and experimental design

Fifty female Sprague-Dawley (SD) rats were sourced from Nanjing Kaisijia Biotechnology Co., Ltd. They were housed at the Animal Center of the Jiangsu Province Institute of Traditional Chinese Medicine under standard conditions (23°C ± 2°C temperature, 50% ± 5% humidity, and a 12-h light/dark cycle) for 7 days. All animal experimental procedures were in strict compliance with European community guidelines and approved by the Ethics Committee of Jiangsu Province Institute of Traditional Chinese Medicine (Permission No. AEWC-20220615-214).

In this research, we utilized corticosterone to establish a POI model (Miao et al., 2020). The rats were randomly divided into five groups (n = 10/group): normal control (NC), model control (MC), Progynova treatment (PT) as the positive control, and two BHF treatment groups with low (L-BHF) and high (H-BHF) dosages. The rats of NC received subcutaneous saline injections, while the rest were administered corticosterone (40 mg/kg, w/w) for 3 weeks to induce POI. Since the first day of modeling, rats were given drugs administration at a volume of 10 mL/kg. The NC and MC groups were received saline plus 0.5% carboxymethylcellulose, while rats in the PT and BHF treatment groups were orally administered PT (10 mg/mL) or BHF (L-BHF: 171.5 mg/mL of lyophilized powder, H-BHF: 686 mg/mL of lyophilized powder) once daily for 3 weeks. The BHF dosage was selected based on prior research (Miao et al., 2020). And all subsequent experiments were only carried out after the treatment had been completed.

### 2.4 Assessment of ovarian function

Body weight was monitored weekly during the experiment. At its conclusion, tissue and blood samples were collected for analysis. Serum levels of corticosterone, FSH, LH, AMH and E_2_ were measured using ELISA kits per the manufacturer’s instructions. Ovaries were rapidly excised, washed with PBS, and weighed to calculate the ovarian index (ovarian weight/body weight). Sections of the ovaries were fixed in 4% paraformaldehyde for a minimum of 24 h, embedded in paraffin, sectioned into 5 μm slices, and stained with Hematoxylin and Eosin (H&E) to assess follicular changes.

### 2.5 Cells culture and viability assay

The human ovarian granulosa cell line (KGN cells) was obtained from EK Bioscience (Shanghai, China) and cultured in DMEM/F-12 medium (Jiangsu KeyGEN BioTECH Corp., Ltd., China) with 10% FBS (Gibco, United Kingdom) in a 37°C, 5% CO_2_ incubator (Thermo Fisher Scientific, Waltham, MA, United States). KGN cells in the logarithmic growth phase were plated in 96-well plates at a density of 8 × 10^3^ cells/well for 12 h. The cells were then incubated with varying concentrations of palmitic acid (PA, 50, 100, 200 μM), BHF (200, 400 μg/mL), or a combination of PA and BHF for 24 h. Subsequently, 10 μL MTT solution was added to each well and incubated at 37°C for 4 h. After discarding the medium, 100 μL DMSO was added to each well. Cell viability was determined at 490 nm using a microplate reader (SpectraMax^®^ i3x, Molecular Devices, United States) and calculated as follows: cell viability (%) = [OD 490 (sample)/OD 490 (control)] × 100%.

### 2.6 E_2_ Enzyme-linked immunosorbent assay

KGN cells were seeded in 24-well plates at a density of 3 × 10^4^ cells/well for 12 h, followed by treatment with PA, with or without BHF (200, 400 μg/mL), under FSH (50 ng/mL) stimulation for 24 h. Culture supernatants were then collected, and E_2_ concentrations were measured using the E_2_ Human ELISA Kit according to the manufacturer’s instructions. E_2_ levels were determined based on standard curves.

### 2.7 Oil red O and Nile Red staining

Ovarian tissues were fixed in 4% paraformaldehyde, embedded in Tissue-Tek OCT, and sectioned into 5-μm-thick slices for Oil Red O staining. Neutral lipids were visualized and imaged using an inverted microscope (Leica DM 11, Germany).

For Nile Red staining of KGN, cells were seeded into 6-well plates and treated with PA (50, 100, 200, 400 μM) or PA (200 μM) combined with or without BHF (200, 400 μg/mL) for 24 h. Post-treatment, cells were washed with PBS, fixed with 4% paraformaldehyde for 10 min, and stained using a Nile Red staining kit. After being rinsed twice with PBS, lipid droplets were visualized and photographed as mentioned above.

### 2.8 Immunohistochemistry

Immunohistochemistry was performed on paraffin-embedded sections using the avidin-biotin complex method (Zhao et al., 2021). Slides were incubated overnight at 4°C with primary antibodies against PCNA. Subsequently, biotinylated anti-rabbit IgG secondary antibodies and DAPI (Beyotime, China) were applied in sequence, with protection from light. Images were captured using a light microscope (Leica DM 11, Germany) and analyzed with Image-Pro Plus 6.0 software (Media Cybernetics, Maryland, United States).

### 2.9 Immunofluorescence

For immunofluorescence, sections from paraffin-embedded tissues were stained, followed by TUNEL and ROS staining according to standard protocols. GC cells were also fixed and stained with TUNEL and ROS. Images were acquired using a Nikon TS2R-FL fluorescent inverted microscope and quantified with Image-Pro Plus 6.0 software (Media Cybernetics, Maryland, United States).

### 2.10 Immunoblot analysis

Ovarian tissue and KGN cells were lysed using RIPA buffer supplemented with protease and phosphatase inhibitors. Protein concentrations were quantified using a BCA kit, and equal amounts of protein (30 μg/lane) were separated by SDS-PAGE, then transferred onto nitrocellulose filters. The membranes were blocked with 5% skimmed milk for 2 h and incubated with specific primary antibodies overnight at 4°C. After washing with TBST, membranes were incubated with horseradish peroxidase-conjugated secondary antibodies. Protein bands were visualized using ChemiScope S6 (Clinx Science Instruments Co., Ltd., Shanghai, China) and analyzed using ImageJ software. The primary and secondary antibodies used were: anti-PPARγ (Mouse, Proteintech, 1:1,000), anti-c/EBPα (Rabbit, Proteintech, 1:1,000), anti-ATGL (Rabbit, Proteintech, 1:1,000), anti-CPT1 (Mouse, Proteintech, 1:1,000), anti-LC3B (Rabbit, ABclonal, 1:1,000), anti-p62 (Rabbit, ABclonal, 1:1,000), anti-GAPDH (Mouse, Proteintech, 1:2,000), and anti-β-actin (Rabbit, Beyotime, 1:2,000).

### 2.11 Statistical analyses

Data were presented as mean ± SEM. Statistical analyses were conducted using Prism 8.4.0 (GraphPad Software, Inc., United States). Normality and homogeneity of variance tests were respectively performed with Shapiro-Wilk and Brown-Forsythe tests before proceeding with the statistical analysis (both *p* > 0.05 in the present study. A One-Way ANOVA with a subsequent LSD test was applied for multiple group comparisons, or an independent samples t-test for comparisons between two groups. A *p*-value < 0.05 was considered statistically significant.

## 3 Results

### 3.1 Chemical components analysis of BHF

**FIGURE 1:**
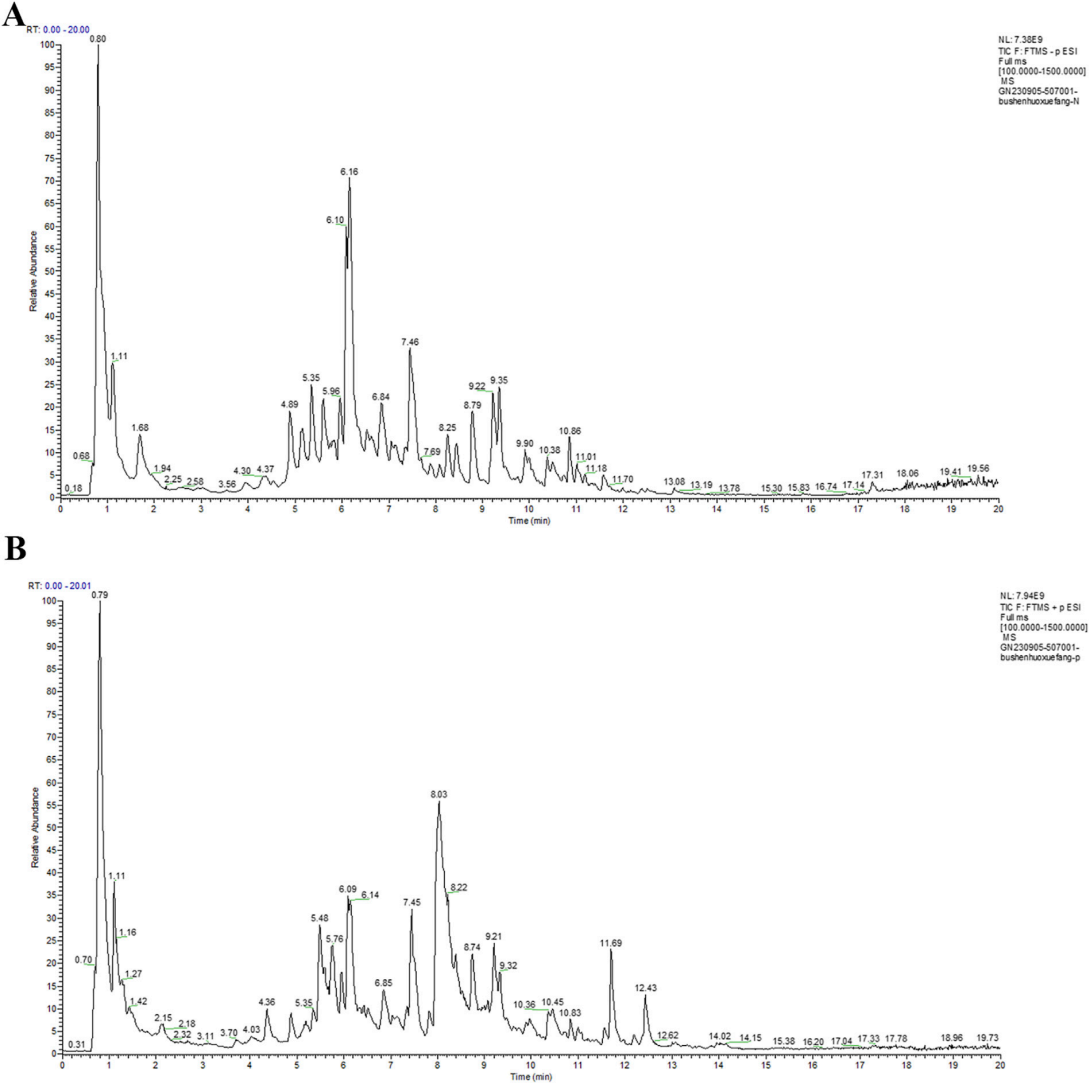
UPLC-MS/MS TICs of BHF in negative-ion mode **(A)** and positive-ion mode **(B)**.

UPLC-MS/MS was utilized to identify the chemical components of BHF extract. The total ion chromatograms (TIC) of BHF are depicted in Figures 1A, B. Using Compound Discoverer 3.3, along with mzCloud and mzVault databases, 350 ingredients were identified in BHF. The top 23 compounds are enumerated in Table 2.

**TABLE 2:** Chemical components within BHF extract by UHPLC-MS/MS.

No.	Rt/min	Molecular formula	Experimental m/z	Error/ppm	Identification	Contents (μg/g)
1	0.78	C_6_H_14_N_4_O_2_	174.1117 [M + H]^+^	−0.15	DL-Arginine	1441.10
2	0.79	C_12_H_22_O_11_	342.1162 [M-H]^−^	0.08	Sucrose	8268.25
3	0.81	C_7_H_12_O_6_	192.0627 [M-H]^−^	−3.58	Quinic acid	7012.78
4	1.11	C_6_H_8_O_7_	192.0264 [M-H]^−^	−3.24	Citric acid	11864.68
5	1.69	C_7_H_6_O_5_	170.0207 [M-H]^−^	−4.82	Gallic acid	11266.87
6	4.89	C_25_H_28_O_16_	584.1381 [M-H]^−^	0.57	Neomangiferin	7748.95
7	5.12	C_23_H_28_O_12_	496.1584 [M-H]^−^	0.66	Oxypaeoniflorin	3075.01
8	5.17	C_16_H_18_O_9_	354.0953 [M-H]^−^	0.49	Chlorogenic acid	2843.68
9	5.36	C_17_H_20_O_9_	368.1107 [M-H]^−^	−0.22	Methyl chlorogenate	11271.79
10	5.60	C_19_H_18_O_11_	422.0851 [M-H]^−^	0.51	Isomangiferin	11832.60
11	5.75	C_9_H_10_O_2_	150.0679 [M + H]^+^	−1.32	Ethyl benzoate	2084.84
12	6.09	C_17_H_20_O_9_	368.1109 [M-H]^−^	0.56	4-O-feruloyl-D-quinic acid	27220.64
13	6.15	C_38_H_52_N_6_O_8_S_4_	848.2745 [M-H]^−^	1.82	(2S,2′S)-2,2′-{Disulfanediylbis [2,1-ethanediylimino (1-oxo-2,1-ethanediyl) (3S)-3,4-dihydroisoquinoline-2,3(1H)-diylcarbonylimino]bis [4-(methylsulfanyl)butanoic acid]	5481.35
14	6.17	C_23_H_28_O_11_	480.1633 [M-H]^−^	0.29	Paeoniflorin	22198.34
15	6.33	C_16_H_16_N_2_O_6_	332.1009 [M-H]^−^	0.11	2,6-DIMETHYL-5-METHOXYCARBONYL-4-(3-NITROPHENYL)-1,4-DIHYDROPYRIDINE-3-CARBOXYLIC ACID	5265.57
16	7.47	C_45_H_76_O_19_	920.4990 [M-H]^−^	0.95	Trigoneoside Xb	9074.25
17	8.45	C_39_H_50_O_19_	822.2959 [M-H]^−^	1.59	Epmedin C	3276.76
18	8.78	C_33_H_40_O_15_	676.2377 [M-H]^−^	1.42	Icariin	6385.02
19	9.22	C_45_H_74_O_18_	902.4884 [M-H]^−^	0.94	(3beta,5beta,25S)-26-(beta-D-Glucopyranosyloxy)furost-20 (22)-en-3-yl 2-O-beta-D-glucopyranosyl-beta-D-galactopyranoside	5192.29
20	9.36	C_30_H_32_O_12_	584.1900 [M-H]^−^	1.03	Benzoylpaeoniflorin	7935.29
21	9.90	C_18_H_34_O_5_	330.2407 [M-H]^−^	0.32	(15Z)-9,12,13-Trihydroxy-15-octadecenoic acid	4306.41
22	11.70	C_12_H_16_O_2_	192.1149 [M + H]^+^	−0.6	Senkyunolide A	893.046
23	12.44	C_12_H_14_O_2_	190.0992 [M + H]^+^	−0.77	Ligustilide	698.54

### 3.2 BHF alleviated ovarian function in POI rats

**FIGURE 2:**
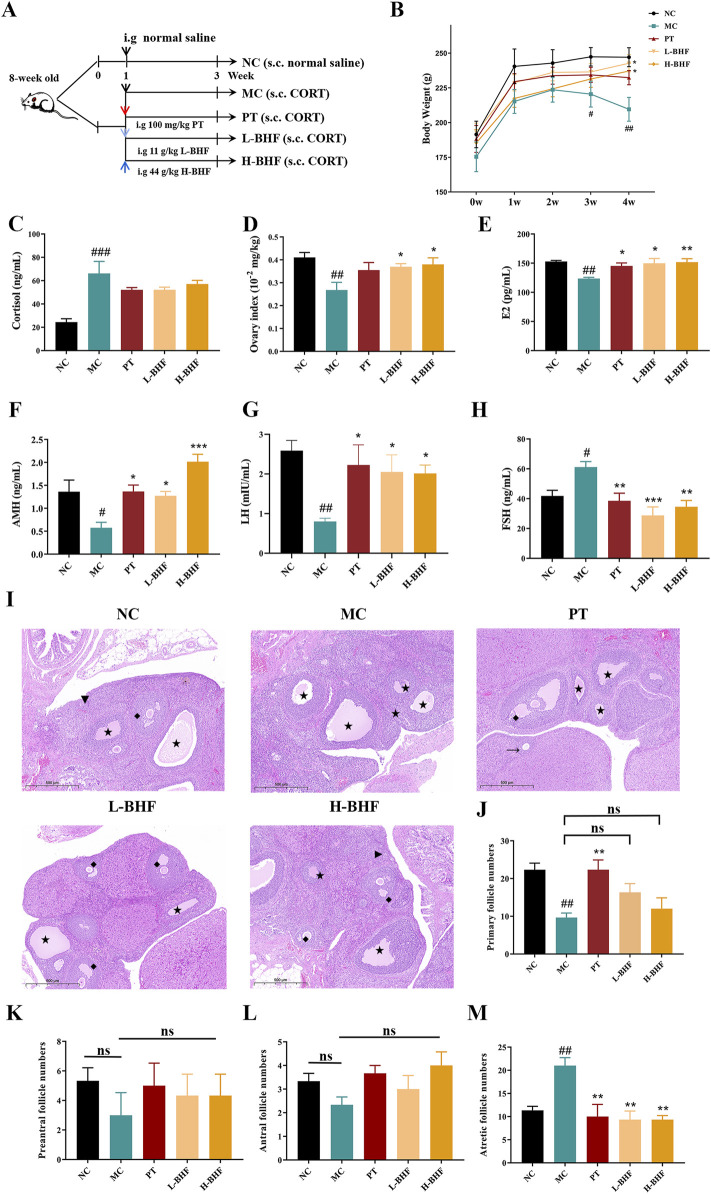
BHF’s impact on body weight and ovarian function in CORT-induced POI rats: **(A)** Study design workflow. **(B)** Body weight over the weeks. **(C)** Serum cortisol levels. **(D)** Ovarian index (ovarian weight/body weight). **(E–H)** Serum levels of E_2_, AMH, LH, and FSH. **(I)** H&E staining ovarian micrographs. **(J–M)** Numbers of primary follicles (Triangle), preantral follicles (arrow), antral follicles (rhomboid), and atretic follicles (Five-pointed star). Data are expressed as mean ± SEM. ^#^ *p* < 0.05, ^##^ *p* < 0.01, and ^###^ *p* < 0.001 vs. the NC group. *p < 0.05, **p < 0.01, and ***p < 0.001 vs the MC group. NC, normal control. MC, model control. PT, positive control. L-BHF, BHF treatment with low dosage. H-BHF, BHF treatment with high dosage.

The therapeutic effects of BHF on POI were assessed using 8-week-old female Sprague-Dawley rats, subjected to BHF for 3 weeks under CORT-induced conditions (Figure 2A). Figure 2B shows that the body weights in the MC group decreased significantly compared to the NC group, whereas BHF treatment notably increased body weight at the end of the experiment. Serum corticosterone levels (Figure 2C), indicative of stress response, were significantly higher in the MC group than in the NC group. However, BHF administration reduced these changes. Ovarian function assessments revealed that ovary index, E_2_, AMH, and LH levels were considerably lower in the MC group compared to the NC group. BHF intervention notably elevated these parameters (Figures 2D–G). BHF treatment also reversed elevated FSH levels in POI rats (Figure 2H). Ovarian morphology, evaluated by H&E staining (Figure 2I), showed that MC rats had an increased number of atretic follicles, while BHF treatment significantly reduced them (Figure 2M). We found that the numbers of primary, preantral, and antral follicles were also exhibited a trend of increasing in BHF-treated POI rats, although there were no significant differences (Figures 2J–L). These findings suggest that BHF restored hormone synthesis and reduced excessive follicular atresia, thereby maintaining ovarian function.

### 3.3 BHF alleviated proliferation inhibition and apoptosis in rat ovary tissues

**FIGURE 3:**
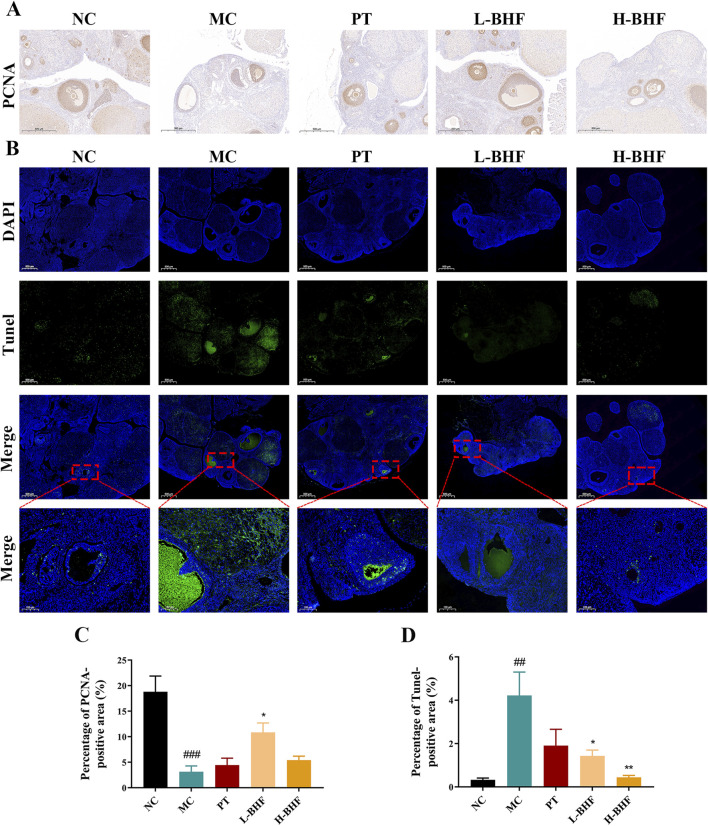
Proliferation and apoptosis in rat ovarian sections using PCNA and TUNEL staining across all groups. **(A)** Immunohistochemistry staining of PCNA (magnification, ×100). **(B)** Immunofluorescence staining of TUNEL (magnification, ×100). **(C, D)** Quantitative analysis of positive areas in the ovaries. Data were expressed as mean ± SEM. ^##^ *p* < 0.01 and ^###^ *p* < 0.001 vs the NC group. *p < 0.05 and **p < 0.01 vs the MC group. NC, normal control. MC, model control. PT, positive control. L-BHF, BHF treatment with low dosage. H-BHF, BHF treatment with high dosage.

Proliferation and apoptosis in follicles were evaluated using PCNA and TUNEL staining, respectively. Figure 3 illustrates that the proportion of PCNA-positive areas in follicles was significantly reduced in the MC group compared to the NC group, but markedly increased following BHF treatment. Conversely, TUNEL staining revealed increased apoptosis in the MC group relative to the NC group. BHF intervention notably lessened this effect. These findings indicate that BHF effectively reduced both proliferation inhibition and apoptosis in GCs within follicles.

### 3.4 BHF modulated lipid metabolism, alleviated ROS and excessive autophagy in POI rats

**FIGURE 4:**
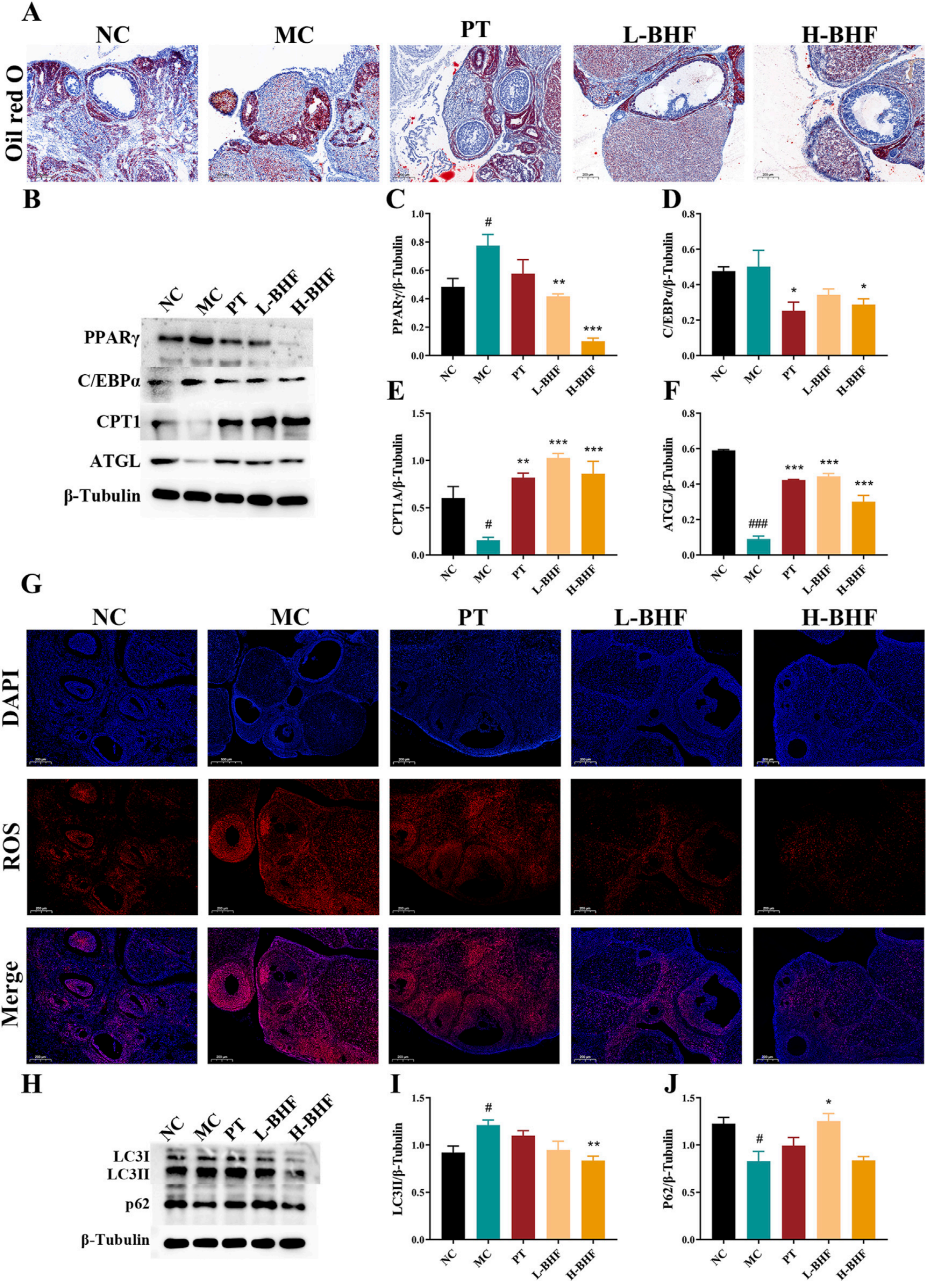
BHF improved lipid metabolism, ROS and autophagy in POI rats **(A)** Oil Red O staining (magnification ×100). **(B)** Expression levels of PPARγ, c/EBPα, CPT1A and ATGL. **(C–F)** Semi-quantitative analysis of PPARγ, c/EBPα, CPT1A and ATGL. **(G)** ROS staining (magnification ×100). **(H)** Expression levels of LC3 and P62. **(I, J)** Semi-quantitative analysis of LC3 and P62. Data were expressed as mean ± SEM. ^#^ *p* < 0.05 and ^###^ *p* < 0.001 vs the NC group. *p < 0.05, **p < 0.01 and ***p < 0.001 vs the MC group. NC, normal control. MC, model control. PT, positive control. L-BHF, BHF treatment with low dosage. H-BHF, BHF treatment with high dosage.

To assess the impact of BHF on lipid accumulation, ROS, and autophagy, ovarian lipid deposition levels were first examined using Oil Red O staining (Figure 4A). BHF treatment significantly decreased lipid accumulation in the ovaries of POI rats. Further investigation into lipid metabolism-related proteins (Figures 4B–F) showed that BHF therapy reduced lipogenesis protein expression (PPARγ and C/EBPα), increased lipolysis protein levels (ATGL), and boosted fatty acid β-oxidation protein expression (CPT1A). ROS levels and autophagy status in the ovaries were also evaluated post-BHF treatment. Results indicated that ROS levels and LC3II expression were substantially higher in the MC group compared to the NC group, while significantly reduced after BHF treatment. In contrast, P62 expression, markedly decreased in the MC group, was restored with BHF administration (Figures 4G–H). Overall, these data demonstrate that BHF treatment reduced lipid accumulation, ROS production, and excessive autophagy.

### 3.5 BHF mitigated PA-induced KGN cells injury via regulating lipid metabolism

**FIGURE 5:**
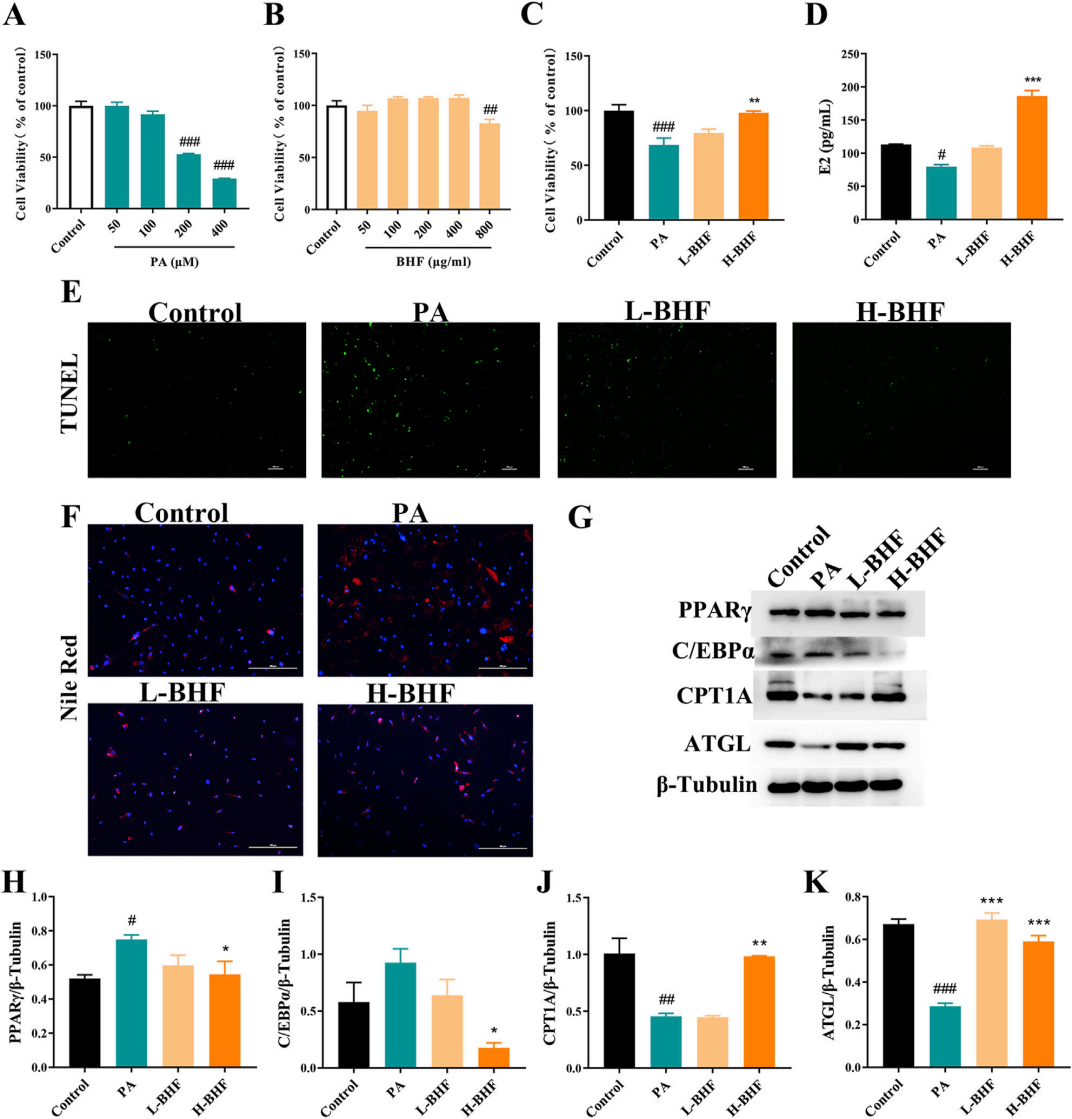
BHF improved lipid metabolism, ROS and autophagy in KGN cells. **(A–C)** Cell viability assays. **(D)** E_2_ levels assays. **(E, F)** TUNEL and Nile Red staining (magnification ×100). **(G)** Expression levels of PPARγ, c/EBPα, CPT1A and ATGL. **(H–K)** Semi-quantitative analysis of PPARγ, c/EBPα, CPT1A and ATGL. Data were expressed as mean ± SEM. #*p* < 0.05 and ###*p* < 0.001 vs the NC group. *p < 0.05, **p < 0.01 and ***p < 0.001 vs the MC group.

To understand how BHF diminishes the injury of GCs, a lipid-loading KGN cell model was established using PA. Figure 5A demonstrates that 24-h incubation with PA (50, 100, 200, and 400 μM) significantly decreased cell viability in KGN cells in a dose-dependent manner. Consequently, PA at 200 μM for 24 h was selected for subsequent experiments. BHF cytotoxicity was then evaluated in KGN cells. Results indicated that up to 400 μg/mL of BHF did not exhibit cytotoxic effects (Figure 5B). The impact of BHF at 200 and 400 μg/mL on PA (200 μM) stimulated KGN cells was further examined. Figures 5C–E show that BHF treatment not only significantly improved cell viability and E_2_ levels but also notably reduced apoptosis in PA-induced KGN cells. Additional analysis revealed that BHF decreased PA-induced lipid accumulation (Figure 5F). PA treatment increased PPARγ and C/EBPα expression, which was negated following BHF treatment. Expectedly, ATGL and CPT1A expression markedly decreased with PA treatment but were substantially reversed upon co-incubation with BHF (Figures 5G–K). Collectively, these findings indicate that BHF protects GCs by regulating lipid metabolism, thereby reducing lipid accumulation.

### 3.6 BHF inhibited ROS accumulation and autophagy in PA-induced KGN cells

**FIGURE 6:**
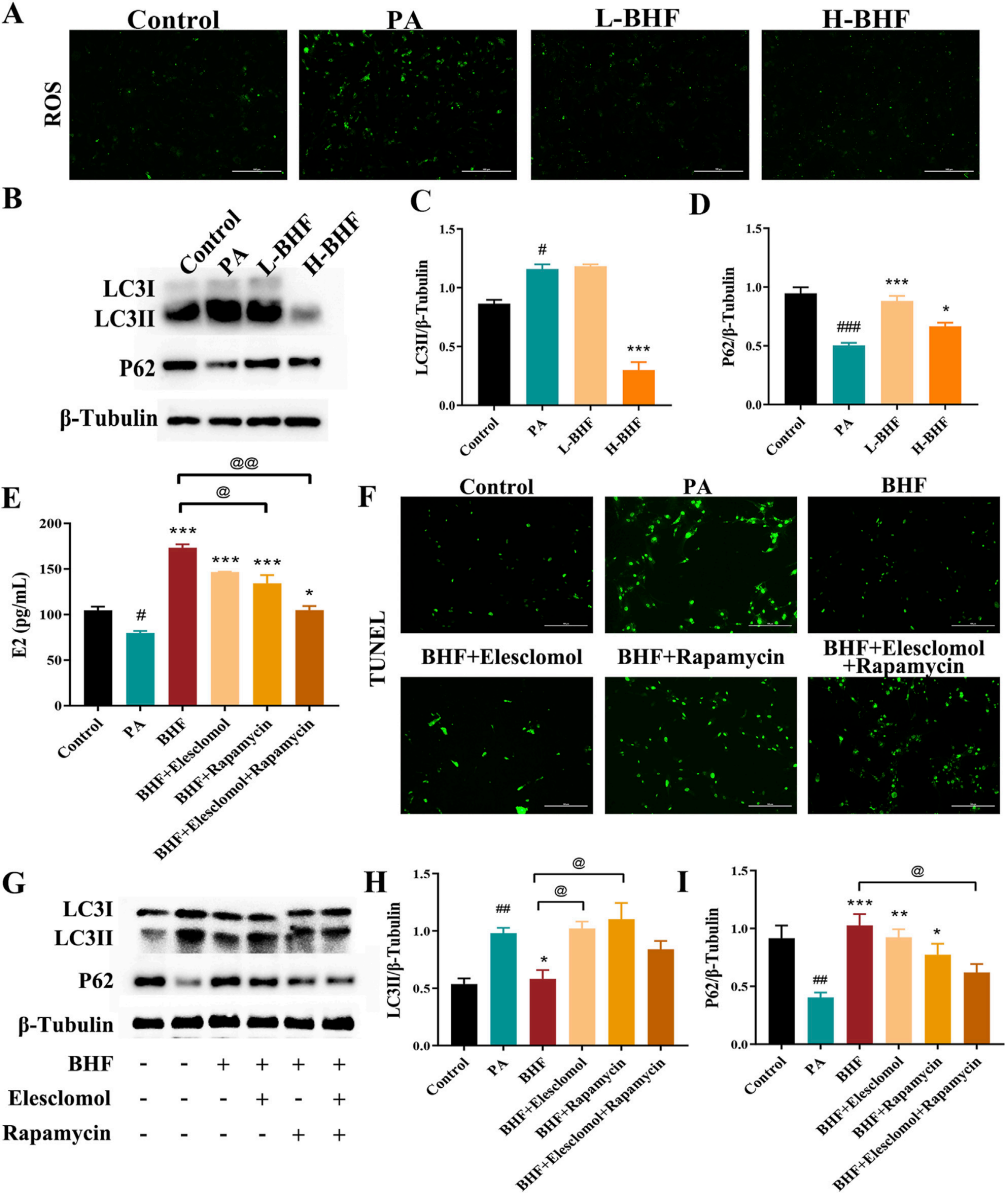
BHF inhibited ROS and autophagy in KGN cells. **(A)** ROS staining (magnification ×100). **(B)** Expression levels of LC3 and P62. **(C, D)** Semi-quantitative analysis of LC3 and P62. **(E)** E_2_ levels assays. **(F)** TUNEL staining (magnification ×100). **(G)** LC3 and P62 expression levels with and without elesclomol or rapamycin addition. **(H, I)** Semi-quantitative analysis of LC3 and P62 with and without elesclomol or rapamycin addition. Data were expressed as mean ± SEM. ^#^ *p* < 0.05 and ^###^ *p* < 0.001 vs the NC group. *p < 0.05 and ***p < 0.001 vs the MC group. ^@^ *p* < 0.05 and ^@@^ *p* < 0.05 vs the BHF group.

The effect of BHF on ROS and autophagy was evaluated in PA-stimulated KGN cells. Figures 6A–C shows that 24-h BHF treatment significantly reduced ROS levels and LC3II expression. The same treatment also increased p62 expression in PA-stimulated KGN cells (Figures 6B, D). Our data demonstrated that BHF was most effective at a concentration of 400 μg/mL, which was used for further experiments. The effects of BHF on E_2_ levels, apoptosis, ROS, and autophagy were partially negated by treatment with elesclomol (a ROS inducer) and rapamycin (an autophagy inducer). Co-incubation with elesclomol and rapamycin almost entirely counteracted the effects of BHF under model conditions (Figures 6E–I). In conclusion, BHF was found to inhibit cell apoptosis and enhance E_2_ secretion by reducing ROS accumulation and hyperactivation of autophagy.

## 4 Discussion

POI, a major factor contributing to infertility, significantly affects the quality of life in women. Currently, there are no specific, clinically proven treatments for POI. However, recent studies suggest that Traditional Chinese Medicines offer promising benefits in managing POI (Xiu et al., 2023; Zhou et al., 2021). BHF, a formula based on clinical experience, has shown protective effects against POI (Miao et al., 2020). Yet, its underlying mechanism remains to be fully understood. In this study, we found that BHF effectively countered hormonal imbalances (E_2_, LH, FSH, and AMH) and reduced follicular depletion in POI rats. Both *in vivo* and *in vitro* experiments indicated that BHF reduced proliferation inhibition and apoptosis in GCs by decreasing lipid deposition-induced ROS accumulation and autophagy.

BHF comprises eleven traditional Chinese medicinal herbs and has complex chemical constituents. This research identified the top 23 compounds in BHF (Table 2) with pharmacological activities like lipid-lowering, insulin sensitization, antithrombotic, anti-inflammatory, and antioxidant effects (Jang et al., 2017; Ma et al., 2017; Zhang et al., 2014). Notably, paeoniflorin, isomangiferin, sucrose, icariin, benzoylpaeoniflorin, neomangiferin, and chlorogenic acid are known lipid metabolism regulators (Bi et al., 2023; Geidl-Flueck et al., 2021; Ma et al., 2017; Shao et al., 2021; Xu et al., 2021; Zhou et al., 2015). Our previous proteomic screening indicated that differentially expressed proteins in BHF-treated POI rats primarily involved triglyceride homeostasis and cholesterol metabolism (Miao et al., 2020). Recent studies have also highlighted that cholesteryl ester and triacylglycerol are key metabolites in diminished ovarian reserve rats treated with BHF (Zeng et al., 2023). Therefore, BHF’s efficacy in alleviating POI may be attributed to its lipid-regulating properties.

Lipids are recognized as crucial for ovarian function maintenance. Research indicates that lipids not only provide energy to oocytes but also serve as precursors for steroid hormones synthesized in granulosa and theca cells (Liu et al., 2022). However, lipid accumulation can cause significant ovarian damage. Previous studies have demonstrated that excessive adipose tissue accelerates ovarian follicle loss in rats, leading to premature ovarian failure (Nteeba et al., 2014). Additionally, FFA accumulation in ovaries correlates with oocyte mitochondrial dysregulation and apoptosis in cumulus-oocyte complexes, contributing to ovarian function depletion (Wu et al., 2022). Lipid-lowering agents, such as rosuvastatin, curcumin, and resveratrol, have shown protective effects on ovarian function in POI models (Elkady et al., 2019; Said et al., 2016; Yan et al., 2018). In this study, lipid deposition in POI rat ovaries and PA-induced lipid accumulation leading to reduced E_2_ secretion and increased apoptosis in GCs were observed. These effects were reversed with BHF treatment, suggesting that the efficacy of BHF against POI is linked to its lipid accumulation reduction. Further, BHF regulated lipid metabolism by inhibiting lipid synthesis (PPARγ and c/EBPα expression), enhancing lipid catabolism (ATGL expression), and boosting lipid oxidation (CPT1A expression). Thus, BHF treatment in POI might be related to reprogramming lipid metabolism to alleviate lipid accumulation. Notably, premature menopause, especially in POI patients, increases cardiovascular risk factors such as weight gain, visceral adiposity, and lipid abnormalities (van Lennep et al., 2016; van Lennep et al., 2023). The 2019 ACC/AHA guideline also considers premature menopause (before age 40 years) a risk-enhancing factor for atherosclerotic cardiovascular disease, recommending statin treatment for women at borderline or intermediate risk (Arnett et al., 2019). Therefore, combining effective POI treatments with lipid-lowering drugs may be a novel strategy for future POI management.

Moreover, disrupted lipid metabolism leads to elevated ROS levels, implicated in conditions like fatty liver disease and colon cancer (Abulikemu et al., 2023; Shi et al., 2022). Previous studies have established that excessive ROS can cause oxidative damage, triggering atresia in follicles and eventually resulting in premature ovarian failure (Agarwal et al., 2012). Our data indicate that BHF treatments significantly reduced ROS accumulation in corticosterone-induced rats and PA-challenged GCs. The use of elesclomol, a ROS inducer, partially reversed the effects of BHF in PA-challenged GCs. These findings suggest that BHF improves POI by reducing lipid-induced ROS accumulation.

Autophagy is crucial for maintaining cellular homeostasis by eliminating misfolded proteins or defective organelles. However, its hyperactivation can lead to the extensive degradation of vital cell components. There is substantial evidence that excessive autophagy induces apoptosis in GCs (Zhou et al., 2019). Duerrschmidt N et al. reported that low-density lipoprotein-induced autophagic death in GCs contributes to follicular atresia (Duerrschmidt et al., 2006). Furthermore, chronic stress is known to accelerate GC senescence, which is linked to ROS-induced autophagy (Sun et al., 2021). Current therapeutic approaches, such as melatonin, hyperoside, and human umbilical cord-derived mesenchymal stem cells, aim to reduce autophagy to improve POI (Dai et al., 2023; Xie et al., 2021; Zhu et al., 2022). Our findings show that BHF reduced the accumulation of LC3II and the decrease in P62 in CORT-induced rats and PA-challenged GCs, thereby inhibiting excessive autophagy. Co-treatment with rapamycin, an autophagy inducer, partially nullified BHF’s anti-apoptotic and E_2_ enhancing effects in PA-induced GCs. Notably, the effects of BHF were almost negated under co-incubation with rapamycin and elesclomol in PA-induced GCs. These results suggest that BHF attenuates lipid-induced autophagy, thereby boosting E_2_ secretion. Conversely, inadequate autophagy can impair GC differentiation and E_2_ synthesis (Shao et al., 2022). Given that autophagy has varied effects on cell proliferation or apoptosis depending on cellular conditions (Mazure and Pouyssegur, 2010), we speculate that the observed differences could be due to the status of GCs arising from the various model establishment methods.

## 5 Conclusion

In summary, our study has shown that BHF has beneficial effects on POI rats by enhancing hormonal balance and reducing follicular depletion. Further analysis indicated that the mechanisms of BHF are closely related to its ability to reduce lipid accumulation-induced ROS and autophagy in ovaries and GCs by altering lipid metabolism. These findings suggest that modulation of lipid metabolism could be a promising therapeutic approach for the treatment of POI. Notwithstanding, our study also exists some limitations. Firstly, the chemical components of BHF were identified in this paper. However, the active ingredients should be screened based on the curative effects of improving POI. Secondly, this experiment only provided preliminary detection of the representative proteins in lipid metabolism and did not carry out a full screening. Finally, this experiment did not probe the upstream regulators of lipid metabolism in the treatment POI. Therefore, the pathways and critical targets of BHF modulation lipid metabolism in POI will be the subject of a future study.

## Data Availability

The original contributions presented in the study are included in the article/supplementary material, further inquiries can be directed to the corresponding authors.

## Abbreviations

BHF, Bushen Huoxue formula; POI, premature ovarian insufficiency; PA, palmitic acid; FSH, follicle-stimulating hormone; AMH, anti-Müllerian hormone; E2, estradiol; LH, luteinizing hormone; GCs, granulosa cells; ROS, reactive oxygen species; TG, triglyceride; FFA, free fatty acid; HRT, hormone replacement therapy.
